# Response: Commentary “The sexualized-body-inversion hypothesis revisited: Valid indicator of sexual objectification or methodological artifact?”

**DOI:** 10.3389/fpsyg.2015.01360

**Published:** 2015-09-08

**Authors:** Alexander F. Schmidt

**Affiliations:** Institute for Health and Behaviour, Health Promotion and Aggression Prevention, University of LuxembourgEsch-Sur-Alzette, Luxembourg

**Keywords:** sexual objectification, person perception, object perception, artifact, inversion effect, gender

Recently, Bernard et al. (2012) reported that a mirror task produced no differences for recognition rates of sexualized female stimuli that had been presented in upright vs. upside down orientations (based on acceptance of the null-hypothesis) whereas recognition rates for sexualized males were better in upright vs. upside down presentations. According to their *sexualized-body-inversion hypothesis* (SBIH) the authors concluded that male stimuli were processed configurally (i.e., person perception, amenable to stimulus presentation orientation) as opposed to female stimuli being perceived analytically (i.e., object perception, unimpaired by presentation mode).

This research has been critized (Tarr, 2013; Schmidt and Kistemaker, 2015). Empirically, we have shown greater asymmetry in female vs. male stimuli to explain the original pattern of results. Utilizing the same design and stimuli subsets from Bernard et al. (2012) we replicated their results but replication failed with stricter experimental control (counterbalancing of the original stimulus subsets) and with a newly developed symmetry-matched stimuli set (Schmidt and Kistemaker, 2015). We concluded that the original effect was dependent on two important boundary conditions in a task vulnerable to symmetry confounds between a) male and female stimuli and b) different stimulus subsets. This interpretation has been challenged (Bernard et al., 2015a).

## Statistical significance vs. effect sizes of stimulus symmetry differences

Bernard et al.'s (2015a) main criticism rests on the fact that all interaction effects involving gender and stimulus orientation on asymmetry indicators (body-axis angles; Schmidt and Kistemaker, 2015; Study 1) were not statistically significant. As is commonly known, statistical significance is a function of sample size, effect size, and statistical power of a test to reject the null hypothesis. Our tests of asymmetry between stimuli subsets were severely underpowered because Bernard et al.'s (2012) stimuli contained only two 12-picture stimuli subsets (observed power for *post-hoc* tests ranged between 0.09 and 0.96, falling well-below the usual threshold of 0.80 for 9/10 comparisons). Moreover, we were asked to use Bonferroni-corrections due to multiple testing. This resulted in a very conservative test strategy (without correction, however, the largest contrast was significant). Therefore, we reported descriptive effect sizes that are better indicators of the hypothesized impact of symmetry issues than statistical significance in case of underpowered comparisons (η^2^s = 0.06 and 0.12; *p*s = 0.12 and 0.24 for the critical omnibus Stimulus Gender × Stimulus Orientation and the Stimulus Gender × Stimulus Orientation × Body-Axis interactions, respectively; Schmidt and Kistemaker, 2015, Study 1, pp. 79–80). Specifically, *post-hoc* contrasts revealed substantially larger asymmetry for inverted vs. upright female stimuli (Cohen's *d*s = 0.89, 0.58, 0.47, 0.29 across different body axes) whereas for male stimuli the pattern—albeit less pronounced—ran into the reversed direction (*d*s = −0.45, −0.31, −0.27, −0.16). Magnitude and opposed directedness of symmetry effects (although being non-significant due to the small picture set) preliminarily corroborate differences across gender and stimulus orientation subsets. These results were not mentioned in Bernard et al.'s (2015a) commentary. Crucially, due to the underpowered nature of these tests acceptance of the null hypothesis is critical due to the high risk of a β-error. Thus, we experimentally tested these hints to symmetry confounds in Study 2 doubling the number of stimuli used (Schmidt and Kistemaker, 2015).

## *Post-hoc* contrasts in recognition rates

Observed statistical power in Bernard et al. (2012) was 0.28 for their non-significant crucial *post-hoc* comparison between upright and inverted females. Accordingly, the onus to prove that statistical power was sufficient to interpret the null hypothesis is on Bernard et al. (2012). We based our conclusions on the alternative hypothesis (showing differences between upright und inverted stimuli). Schmidt and Kistemaker's Study 2 clearly speaks against the SBIH based on omnibus tests across counterbalanced stimuli subsets (i.e., the critical Gender × Stimulus Orientation interaction was non-significant, η^2^ = 0.003, *F* < 1, but was further qualified by interacting with Stimulus Subset, η^2^ = 0.26, *p* < 0.001). Moreover, the same *post-hoc* tests as in Bernard et al. (2012) failed to demonstrate the SBIH-effect (again, with positive evidence for the alternative hypothesis). Bernard et al. (2015a) also neglected that sexual objectification effects emerged for male but not for female stimuli in the stimulus subset they had excluded from their study.

Nevertheless, Bernard et al. (2015a) proposed two specific contrasts that in their view speak against our interpretation: Visually inspecting our data they concluded that *post-hoc* tests of each of the upright vs. inverted female stimuli subsets revealed no significant differences (based on acceptance of the null-hypothesis). However, both proposed contrasts yield a calculated mean difference of *d* = 0.29, *p* = 0.096 and *d* = 0.35, *p* = 0.024 further adding to the positive evidence *against* the SBIH. Taken together with our finding that newly constructed *symmetry-matched stimuli* also yielded positive evidence against the SBIH (Schmidt and Kistemaker, 2015, Figure 3), we consider our results as strong evidence that symmetry confounds are a necessary boundary condition for SBIH-effects.

## Conclusion

We agree with Bernard et al. (2015a) that the results of the degree of sexualization manipulation from Schmidt and Kistemaker (2015) restrict the generalizability of the SBIH but do not relate to target sexualization in the original study's stimuli. Moreover, we acknowledge that Bernard et al. (2015b) recently contributed new data in favor of the SBIH (although based on a very small sample of *N* = 21) using their original stimuli in a counterbalanced design. In summary, hitherto mixed findings are reported based on the original stimuli from Bernard et al. (2012, 2015b) that are in opposition to Schmidt and Kistemaker (2015). Thus, as of yet no replication of the SBIH with independent stimuli exists—certainly not with symmetry-matched stimuli. Hence, the robustness of the SBIH is still at question. We agree with Bernard et al. (2015a) that exact (and we would like to add conceptual) replication studies with sufficient statistical power are needed to elucidate the impact of stimulus symmetry (or other possible stimulus confounds) on the SBIH. More studies on this effect would enable meta-analytic integration to resolve this issue.

## Conflict of interest statement

The author declares that the research was conducted in the absence of any commercial or financial relationships that could be construed as a potential conflict of interest.
